# The Improvement of Density Peaks Clustering Algorithm and Its Application to Point Cloud Segmentation of LiDAR

**DOI:** 10.3390/s24175693

**Published:** 2024-09-01

**Authors:** Zheng Wang, Xintong Fang, Yandan Jiang, Haifeng Ji, Baoliang Wang, Zhiyao Huang

**Affiliations:** State Key Laboratory of Industrial Control Technology, College of Control Science and Engineering, Zhejiang University, Hangzhou 310027, China; zheng_wang@zju.edu.cn (Z.W.); xt_fang@zju.edu.cn (X.F.); ydjiang@zju.edu.cn (Y.J.); hfji@zju.edu.cn (H.J.); wangbl@zju.edu.cn (B.W.)

**Keywords:** density peaks clustering (DPC), clustering, pattern recognition, LiDAR, point cloud segmentation

## Abstract

This work focuses on the improvement of the density peaks clustering (DPC) algorithm and its application to point cloud segmentation in LiDAR. The improvement of DPC focuses on avoiding the manual determination of the cut-off distance and the manual selection of cluster centers. And the clustering process of the improved DPC is automatic without manual intervention. The cut-off distance is avoided by forming a voxel structure and using the number of points in the voxel as the local density of the voxel. The automatic selection of cluster centers is realized by selecting the voxels whose gamma values are greater than the gamma value of the inflection point of the fitted γ curve as cluster centers. Finally, a new merging strategy is introduced to overcome the over-segmentation problem and obtain the final clustering result. To verify the effectiveness of the improved DPC, experiments on point cloud segmentation of LiDAR under different scenes were conducted. The basic DPC, K-means, and DBSCAN were introduced for comparison. The experimental results showed that the improved DPC is effective and its application to point cloud segmentation of LiDAR is successful. Compared with the basic DPC, K-means, the improved DPC has better clustering accuracy. And, compared with DBSCAN, the improved DPC has comparable or slightly better clustering accuracy without nontrivial parameters.

## 1. Introduction

As an important technology of pattern recognition, clustering is a hotspot and has been studied and applied in various fields [1,2,3,4,5,6,7,8,9,10,11,12,13,14], including machine vision [4,5], robot sensing [6,7], life and medical sciences [8,9], astronomy [10,11], and many other fields [12,13]. However, with the rapid development of science and technology, the requirements of the applications become higher and higher. To seek more effective clustering methods is an important work for academic research and practical applications [1,2,3,4,5,6,7,8,9,10,11,12,13,14].

Density peaks clustering (DPC), which is also known as clustering by fast search and find of density peaks (CFSFDP), is a relatively new algorithm proposed by Rodriguez and Laio in Science [15]. This algorithm utilizes the density and a new-defined criterion named delta-distance (delta) to find the high-density peaks. The points with high densities and delta values are assigned as the cluster centers, and then each remaining point is assigned to the same cluster as its nearest neighbor of higher density [15]. DPC has the advantages of recognizing non-spherical data and finding the number of clusters intuitively, which are attractive for researchers.

Although DPC provides an effective clustering approach, there are still some aspects that need to be improved [16,17,18,19,20,21,22,23,24,25,26,27]. The clustering process of DPC is not adaptive. Its internal parameters cannot be adjusted adaptively, and the cluster centers cannot be selected automatically. Up to date, although some achievements have been obtained, the research work on the improvement of DPC is not sufficient yet [16,17,18,19,20,21,22,23,24,25,26,27]. More research work should be undertaken.

This work focuses on a new improved DPC algorithm that can avoid the manual determination of the cut-off distance and select the cluster centers automatically, adaptively realizing the clustering process. Meanwhile, LiDAR (light detection and ranging) is an important device in autonomous vehicles, intelligent robots, environment monitoring, emergency management, intelligent transportation, geomatics, etc. [28,29,30,31,32,33,34], and its point cloud segmentation is a fundamental and important task. In this work, the experimental results on point cloud segmentation of LiDAR are used to verify the effectiveness of the improved DPC.

The innovative idea and main contribution of this work can be summarized as:

- A new effective and automatic clustering algorithm is proposed by improving DPC, and the DPC-based algorithm is introduced to point cloud segmentation, which provides a new choice and useful reference for researchers in the field.
- Compared with DPC, the improved DPC avoids the manually set cut-off distance, simplifies the calculation of local density by forming a voxel structure, and selects cluster centers automatically by calculating the analytical solution of the inflection point of the fitted gamma (γ) curve. Additionally, the improved DPC adopts a new merging strategy to overcome the over-segmentation problem of point clouds.

The rest of the manuscript is organized as follows: Section 2 introduces the related works of DPC. Section 3 describes the improved DPC algorithm. Section 4 illustrates the experimental setup and discusses the experimental results. Section 5 draws the conclusion of the manuscript.

## 2. Related Works

DPC assumes that the cluster centers have higher densities than other data points, and the distances between cluster centers and any data points with higher densities are relatively large [15,16,17,18,19,20,21,22,23,24,25,26,27]. The process of using DPC to realize clustering can be mainly divided into three steps [15,16,17,18,19,20,21,22,23,24,25,26,27]: (1) Calculate the local density $\rho_{i}$ and its delta-distance $\delta_{i}$ which is the distance between the point and the nearest point with a higher density. (2) Select the cluster centers by the local density and the delta-distance. (3) Assign the other points to the cluster centers and complete the clustering process.

To calculate the local density of the ith point $\rho_{i}$, the following two formulas are commonly used [15,16,17,18,19,20,21,22,23,24,25,26,27]:

(1) $$\rho_{i}=\sum_{p\neq i}^{N}\chi \left(d_{ip}-d_{c}\right),$$

(2) $$\rho_{i}=\overset{N}{\underset{p\neq i}{\sum }}\exp\left[-{\left(\frac{d_{ip}}{d_{c}}\right)}^{2}\right],$$

where N is the number of points,$\;d_{ip}$ is the Euclidean distance between the ith point and another point (mark it as the pth point), and $d_{c}$ is the cut-off distance. $\chi \left(d\right)$ is a function that when d<0, $\chi \left(d\right)=1$ and when d≥0, $\chi \left(d\right)=0$. In both Equations (1) and (2), the value of $d_{c}$ should be predetermined manually [16,17,18,19,20,21,22,23,24,25].

The delta-distance $\delta_{i}$ is the distance between the ith point and the nearest point whose density is higher than that of the ith point [15,16,17,18,19,20,21,22,23,24,25,26,27]:

(3) $$\delta_{i}=\underset{p:\rho_{p}>\rho_{i}}{\min}(d_{ip}),$$

For the point of the highest density, its delta-distance is the maximum distance between the point of the highest density and any other points.

To select the cluster centers, decision graph and quantity gamma $\gamma_{i}$ are two approaches [15,16,17,18,19,20,21,22,23,24,25,26,27].

The decision graph is to plot a figure of $\delta_{i}-\rho_{i}$ for each point [15,16,17,18,19,20,21,22,23,24,25,26,27]. With a decision graph, the points whose $\rho_{i}$ and $\delta_{i}$ are relatively high are selected as cluster centers [15,16,17,18,19,20,21,22,23,24,25,26,27].

The quantity gamma $\gamma_{i}$ is defined as [15,17,18,19,20,21,22,23,24,25]:

(4) $$\gamma_{i}=\rho_{i}\delta_{i},$$

**Figure 1:**
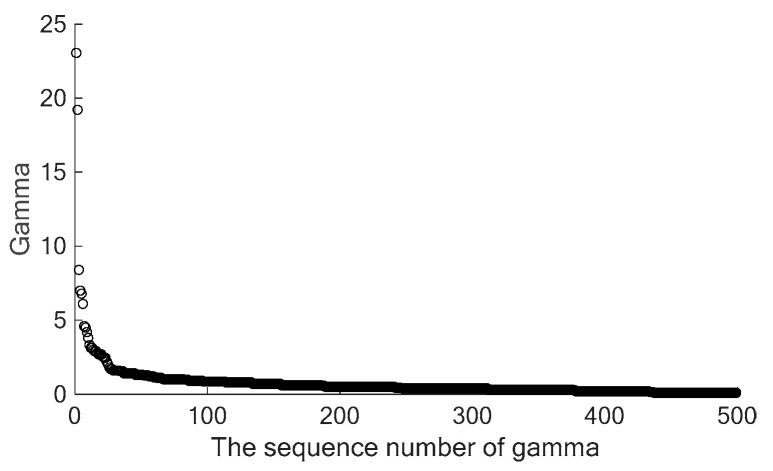
An example of γ curve.

Sort $\gamma_{i}$ in decreasing order, mark it as $\gamma_{n}$ (*n* = 1, 2, …, *N*), and select the points with large $\gamma_{i}$ as cluster centers, which means the points whose products of $\rho_{i}$ and $\delta_{i}$ are large are regarded as cluster centers [15,17,18,19,20,21,22,23,24,25]. Figure 1 shows an example of γ curve. It could be found that the $\gamma_{n}$ decreases sharply when *n* increases. The gamma values $\gamma_{n}$ of most points are small. Only a few points have obviously large gamma values $\gamma_{n}$, and these points with large gamma values are regarded as cluster centers [15,17,18,19,20,21,22,23,24,25]. The rest of points with small gamma values $\gamma_{n}$ are not suitable to be selected as the cluster centers [15,17,18,19,20,21,22,23,24,25].

Finally, the other points are assigned to the cluster centers, and the clustering process is completed. Each unclassified point is assigned to the same cluster as its nearest neighbor with a higher density, and the final clusters are obtained [15,16,17,18,19,20,21,22,23,24,25,26,27].

From the above descriptions, because the cut-off distance $d_{c}$ should be manually predetermined and the cluster centers need to be manually selected, most of the conventional DPC algorithms cannot operate the clustering process automatically and need manual intervention.

To overcome these two drawbacks, researchers have made their efforts: (1) Some researchers try to solve the problem of the manually set cut-off distance. Chen et al. proposed the DHeat method to replace the cut-off distance [16]. Hou et al. used a maximum distance in K-nearest neighbors to compute the local density without the cut-off distance [17]. (2) Some researchers focus on the automatic selection of the cluster centers. Zeng et al. selected the two points with the largest gamma values as the cluster centers of shadow and non-shadow [18]. Yan et al. suggested selecting the points above the decision curve (trained by SVM and Support Vector Regression) as cluster centers [19] or the points above a quadratic curve as cluster centers [20]. Cao et al. introduced an adaptive exponential function curve in the decision graph and selected the points above the exponential function curve as cluster centers [21]. Xu et al. selected the points above the “stairs” of the γ curve as cluster centers [22]. Unfortunately, those cluster center selection methods mentioned above still need to set the cut-off distance manually and to set some decision parameters by human intervention [18,19,20,21,22]. (3) Some researchers try to overcome both the two drawbacks. Liu et al. introduced K-nearest neighbors to calculate the cut-off distance and the local density, automatically select all points whose delta-distance is greater than the cut-off distance as initial cluster centers, and finally merge the clusters if they are density-reachable [23]. In this method, the number of nearest neighbors still needs to be predetermined manually [23]. Wang et al. adopted two criteria (AC1 and AC2) for the automatic selection of cluster centroids and used nonparametric multivariate kernel density estimation to obtain the local density without the cut-off distance [24]. The two criteria need to be set as suggested values, and the d-variate kernel function should satisfy the standard d-variate normal distribution [24].

Based on the above descriptions, it could be found that although significant progress has been made, more research work should be undertaken to overcome these two drawbacks.

## 3. The Improved DPC Algorithm

According to the descriptions in Section 2, to operate the clustering process automatically, in this work, the improvement of DPC focuses on the new approach to obtain the local density $\rho_{i}$ without the predetermination of the cut-off distance $d_{c}$ and the new approach which selects cluster centers automatically.

### 3.1. New Approach to Obtain $\rho_{i}$

Take avoiding the cut-off distance $d_{c}$ and reducing computation into account, we form a voxel structure and use the number of points in the voxel to obtain $\rho_{i}$.

Assume the size of region of interest (ROI) is L×W×H, where *L*, *W*, and *H* are the length, the width, and the height of ROI, respectively. Form a voxel structure in which the voxel is cubic and the side length of the voxel is $l_{s}$. With the voxel structure, the ROI is divided into $N_{v}=N_{x}\times N_{y}\times N_{z}$ cubic voxels, where $N_{x}=L/l_{s}$, $N_{y}=W/l_{s}$, $N_{z}=H/l_{s}$.

The density of the jth voxel is defined as:

(5) $$\rho_{j}=\frac{N_{j}}{V_{j}},$$

where *j* is the index of voxel, $N_{j}$ is the total number of points in the jth voxel, and $V_{j}$ is the volume of the jth voxel. In this work, all voxels have the same volume; $V_{j}$ is regarded as unit volume, i.e., $V_{j}=1$. Meanwhile, the location of the voxel is the coordinates of its center. Further, the delta-distance of the jth voxel $\delta_{j}$ can be determined by Equation (3).

**Figure 2:**
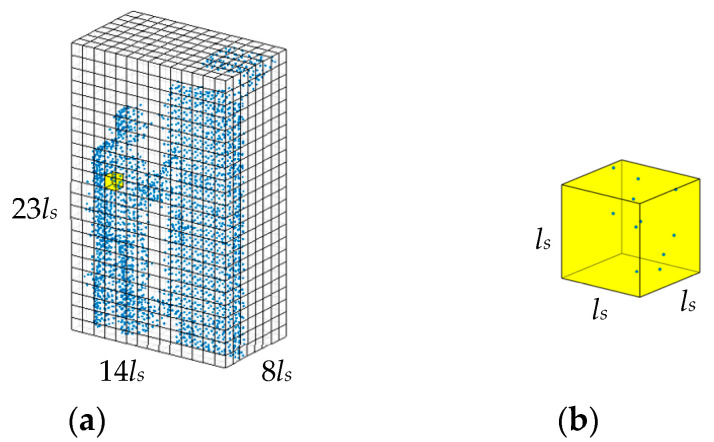
An illustration of voxel structure. (**a**) The whole voxel structure. (**b**) One voxel.

Figure 2 shows an illustration of voxel structure. The size of the ROI is 0.8 m×1.4 m×2.3 m, and the total number of points in the ROI is 2808. The $l_{s}$ of the cubic voxel is 0.1 m. Thus, there are total $N_{v}=8\times 14\times 23$ = 2576 voxels. With Equation (5), it is easy to determine that the density of the voxel shown in Figure 2b is 11. For most practical scenes, most voxels are empty, and we only need to process a small quantity of voxels whose densities are not zero. In the illustration shown in Figure 2, after deleting the empty voxels, the number of voxels for subsequent computation is 499.

From Equation (5), it is clear that we can determine the density of the jth voxel without the cut-off distance $d_{c}$. The predetermination of the cut-off distance $d_{c}$ can be avoided. Furthermore, in the subsequent computation steps (e.g., selection of cluster centers), the operation objects are changed from points to voxels, and only a small quantity of voxels whose densities are not zero need to be processed. Thus, the computation cost could also be significantly reduced.

### 3.2. New Approach to Select Cluster Centers Automatically

The automatic selection of cluster centers is based on $\gamma_{j}$ (Equation (4)). Sort $\gamma_{j}$ in decreasing order, mark it as $\gamma_{m}$, and select the voxels which have larger $\gamma_{m}$ as cluster centers. As mentioned in Section 2, the cluster centers in the γ curve (Figure 1) feature in the two aspects: (1) The $\gamma_{m}$ of cluster centers are large. (2) Only a small quantity of points in the steep part of the γ curve could be regarded as cluster centers, and a mass of points in the flat part of the γ curve are not suitable to be selected as cluster centers.

The γ curve could be fitted to the following function:

(6) $$\gamma =am^{-b},$$

where *a* and *b* are two coefficients, which could be determined by curve fitting. Further, because only a few points (with a larger $\gamma_{m}$) in the steep part of the γ curve could be cluster centers, it is reasonable to find the inflection point of the γ curve and hence to select the voxels whose $\gamma_{m}$ are greater than that of the inflection point as cluster centers.

Obviously, finding the inflection point of the γ curve is the key point. From the viewpoint of mathematics, the so-called inflection point of the curve is the point of maximum curvature of the curve (i.e., the point where the curve bends most sharply) in fact [34].

For the curve described as Equation (6), the point of maximum curvature has an analytical solution. The curvature κ of the fitted curve could be described as [34]:

(7) $$\kappa =\frac{ab\left(b+1\right)m^{-b-2}}{{\left(1+a^{2}b^{2}m^{-2b-2}\right)}^{\frac{3}{2}}},$$

And, the coordinates of the point of maximum curvature ($\tilde{m}$, $\tilde{\gamma }$) are:

(8) $$\tilde{m}={\left(\frac{b+2}{a^{2}b^{2}\left(2b+1\right)}\right)}^{\frac{-1}{2b+2}},$$

(9) $$\tilde{\gamma }=a{\left(\frac{b+2}{a^{2}b^{2}\left(2b+1\right)}\right)}^{\frac{b}{2b+2}},$$

where $\tilde{m}$ is the abscissa of the point of maximum curvature and $\tilde{\gamma }$ is the ordinate of the point of maximum curvature.

After we have obtained the value of $\tilde{\gamma }$, we can select the corresponding voxels whose $\gamma_{m}$ are greater than $\tilde{\gamma }$ as cluster centers. Meanwhile, it is necessary to indicate that to take revealing the inflection/curving characteristics of the γ curve and using less computation cost into account [34,35], in this work, only the first $m_{0}$ $\gamma_{m}$ are used, where $m_{0}$ is the total number of $\gamma_{m}$ that are used for curve fitting.

**Figure 3:**
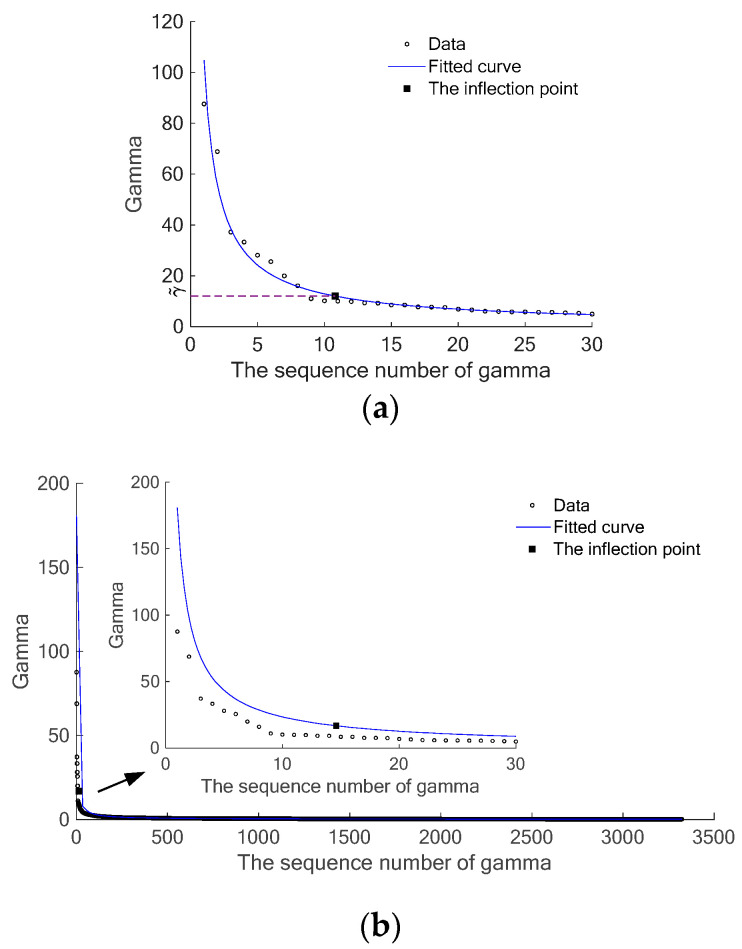
An example of the curve fitting. (**a**) Curve fitting with the first $m_{0}$ $\gamma_{m}$. (**b**) Curve fitting with whole $\gamma_{m}$ and the part of the curve near the inflection point.

Figure 3 shows an example of the curve fitting. In the example, the total number of voxels is 3375, and the number of objects is 6. Figure 3a shows the result of practical curve fitting with the first 30 $\gamma_{m}$ (i.e., $m_{0}=30$), and the inflection point is marked as a solid square. It could be found that the number of initial cluster centers is 8. As a comparison, Figure 3b illustrates the result of curve fitting, which uses the whole $\gamma_{m}$ (i.e., total 3375). The corresponding inflection point is also marked as a solid square, and the number of initial cluster centers is 7. Although both the two results satisfy the clustering requirements, the computation cost of the result in Figure 3b is much greater than that in Figure 3a. Comparing Figure 3a with Figure 3b, it could be found that the fitting result using the first 30 $\gamma_{m}$ is much better than the fitting result using the whole $\gamma_{m}$. The fitting result using the first 30 $\gamma_{m}$ well displays the inflection/curving characteristics of the γ curve. This phenomenon is in accord with the numerical analysis result [35]. As mentioned above, only a small quantity of points in the steep part of the γ curve could be regarded as cluster centers, and the rest, which are a large number of points in the flat part of the γ curve, are not suitable to be cluster centers. Using the whole $\gamma_{m}$, the fitted curve will tend to mainly display the characteristics of the points in the flat part of the γ curve and may not well display the inflection/curving characteristics of the γ curve. Therefore, using the first $m_{0}$ $\gamma_{m}$ not only reduces the computation cost but also better displays the inflection/curving characteristics of the γ curve.

Based on the above discussion, the process of the automatic determination of cluster centers can be described as Algorithm 1, which can be mainly divided into the following five steps:

(1) Calculate $\gamma_{j}$ by Equation (4);
(2) Sort $\gamma_{j}$ in decreasing order and mark it as $\gamma_{m}$;
(3) Fit the γ curve by using the first $m_{0}$ $\gamma_{m}$;
(4) Find the point of maximum curvature and obtain the value of $\tilde{\gamma }$ by Equation (9);
(5) Select the corresponding voxels whose $\gamma_{m}$ are greater than $\tilde{\gamma }$ as cluster centers.

**Algorithm 1:** Automatic determination of cluster centers1: **Input:** local density of voxel $\rho_{j}$, delta-distance $\delta_{j}$ 2: **Output:** cluster centers $\gamma_{c}$ 3: **for** $\rho_{j}>0$ **do** 4:    $\gamma_{j}=\rho_{j}\times \delta_{j}$ 5: **end for** 6: Sort $\gamma_{j}$ in the decreasing order, output $\gamma_{m}$ 7: Fit the $\gamma_{m}-m$ curve where {$m|m\leq m_{0}\}$ 8: Calculate $\tilde{\gamma }$ by Equation (9) 9: $\gamma_{c}=\emptyset$ 10: **for** $\gamma_{m}$ **do** 11:    **if** $\gamma_{m}>\tilde{\gamma }$ **then** 12:      $\gamma_{c}=\gamma_{c}\cup \left\{\gamma_{m}\right\}$ 13:    **else** 14:      **break** 15:    **end if** 16: **end for**


In addition, it is necessary to indicate that for most of the conventional DPC algorithms, the cluster centers are manually selected, i.e., the number of the cluster centers is equal to the number of clustering objects. While, in this work, the cluster centers are selected automatically. To avoid the case of the number of the initial cluster centers less than the number of the clustering objects, this work adopts a conservative way, i.e., selecting the voxels whose $\gamma_{m}$ are greater than $\tilde{\gamma }$ as the initial cluster centers. That is on the basis of the obtained research results and the application experience of DPC [15,17,18,19,20,21,22,23,24,25]. The research works have shown/verified that only a few points located in the steep part of the γ curve could be cluster centers [15,17,18,19,20,21,22,23,24,25]. Therefore, in this work, the demarcation point is the point of maximum curvature. Some points that are located in the curving part of the γ curve and whose $\gamma_{m}$ is greater than the demarcation point are also selected as the cluster centers. That ensures the number of the selected initial cluster centers is greater than the number of clustering objects. The over-segmentation problem will be overcome by the following new merging strategy.

### 3.3. New Merging Strategy

The new merging strategy is to overcome the over-segmentation problem and obtain the final clustering result. The merging process could be mainly divided into three parts. Meanwhile, to reduce the computation cost, for the first two parts of the merging strategy, the operating objects are still non-empty voxels.

(1) Each unclassified voxel is assigned to the same cluster as its nearest neighbor with a higher density;
(2) To overcome the over-segmentation problem, a merging criterion is introduced to determine whether the clusters need to be merged. The merging criterion in this work includes two aspects: (i) If the distance between a voxel in cluster A and any voxel in cluster B is less than a distance $\tilde{d}$, the two voxels are regarded as a neighbor voxel pair. (ii) If the number of neighbor voxel pairs is greater than a number $N_{min}$, the two clusters (cluster A and cluster B) will be merged. In the practical merging process, we do not need to calculate the Euclidean distance between two voxels. We only need to compare the coordinate differences of each dimension between two voxels. If the three coordinate differences are all less than $\tilde{d}$, the two voxels are regarded as a neighbor voxel pair. The $\tilde{d}$ is set as several times of the side length of voxels $l_{s}$. If the number of the neighbor voxel pairs is greater than $N_{min}$, there exist over-segmented clusters that should be merged. Otherwise, the two clusters will not be merged;
(3) The points in each voxel are assigned to the cluster of the corresponding voxel, and the final clustering result is obtained.

**Figure 4:**
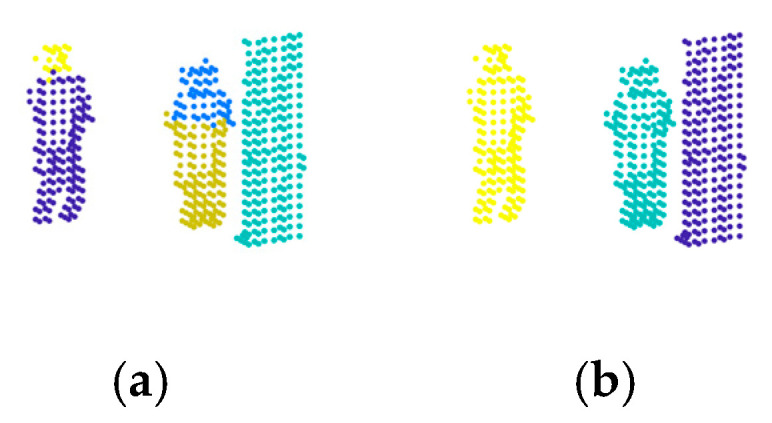
An example of the clustering results. (**a**) The clustering results obtained by the merging strategy of the basic DPC. (**b**) The clustering results obtained by the new merging strategy.

Figure 4 shows an example of the clustering results. The number of objects is 3. As a comparison, Figure 4a shows the clustering results obtained by the merging strategy of the basic DPC [15]. It could be found that the number of clusters is 5, and there still exists over-segmented clusters. Figure 4b shows the clusters obtained by the new merging strategy. In Figure 4b, $\tilde{d}$ is set as 2 times the side length of voxels $l_{s}$. $N_{min}$ is set as 5. It could be found that the number of clusters is 3. The proposed merging strategy is effective, which can effectively merge the over-segmented clusters.

After the merging strategy, the potential over-segmented clusters are merged, and the clustering results become more reasonable.

### 3.4. The Flow Chart of the Improved DPC Algorithm

**Figure 5:**
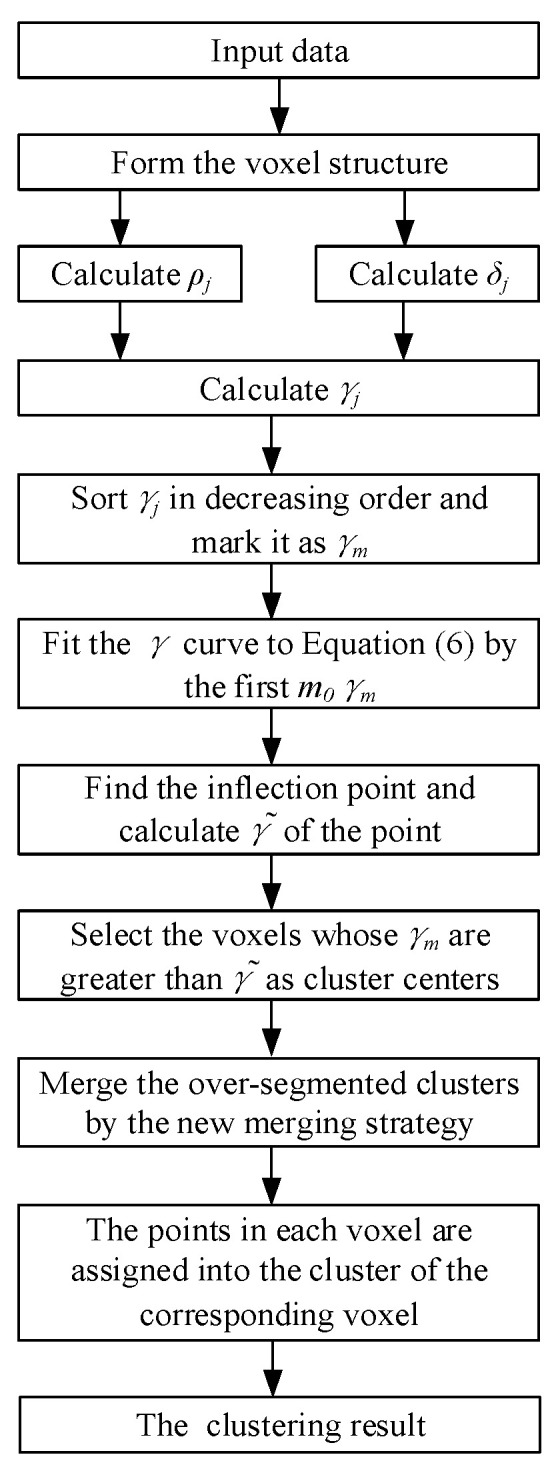
The flowchart of the improved DPC algorithm.

Figure 5 shows the flowchart of the proposed point cloud segmentation method. Firstly, based on the point cloud data obtained by LiDAR, the voxel structure is formed, and the local density $\rho_{j}$, the delta-distance $\delta_{j},$ and the quantity $\gamma_{j}$ of voxels are calculated. Secondly, $\gamma_{j}$ is sorted in decreasing order and marked as $\gamma_{m}$, and the γ curve is obtained. Then, according to Equation (6), the γ curve is fitted using the first $m_{0}$ $\gamma_{m}$. The $\tilde{\gamma }$ of the inflection point, i.e., the point of maximum curvature, is calculated, and the voxels whose $\gamma_{m}$ are greater than $\tilde{\gamma }$ are regarded as cluster centers. Finally, the new merging strategy is adopted to merge the over-segmented clusters, and the point cloud segmentation result is obtained.

In summary, compared with DPC, the improved DPC shows improvements in three aspects: (1) In the traditional DPC algorithms, local density is calculated by Equation (1) or Equation (2), which needs to manually set the cut-off distance and is time-consuming. In the improved DPC, voxel structure is formed to avoid the cut-off distance, and the local density can be directly obtained by the number of points in a voxel. (2) In the traditional DPC algorithms, the cluster centers are defined as the points whose γ (or both ρ and δ) are relatively high. There is no strict definition of “relatively high”, and the number of cluster centers is set manually by a constant number or based on the separability. The improved DPC uses the characteristics of the γ curve to find the “relatively high” points automatically, which is a good attempt based on mathematics. (3) The traditional DPC algorithms may have the over-segmentation problem for point cloud segmentation, though the number of clusters is set as the ideal number. In the improved DPC, a new merging approach is proposed to overcome the potential over-segmentation problem.

## 4. Experiment and Discussion

### 4.1. The Experimental Setup

**Figure 6:**
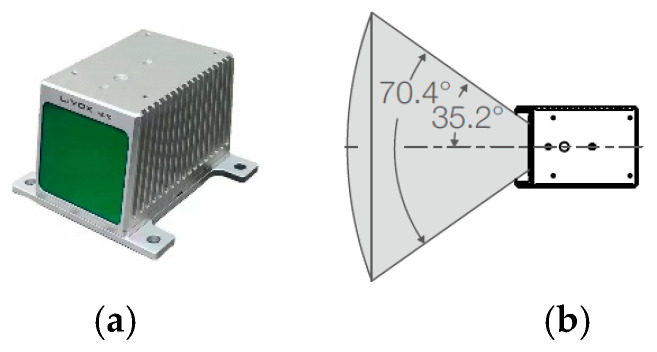
LiDAR MID-70. (**a**) The photo of MID-70. (**b**) The FoV of MID-70.

To verify the effectiveness of the new improved DPC algorithm, experiments were carried out under different scenes in the real world. The LiDAR was MID-70, Livox, Dajiang Innovation Technology (Shenzhen, China), as shown in Figure 6a. The Field of View (FoV) of the LiDAR was 70.4°, as shown in Figure 6b. The computer was DELL G15-5511, Dell Technologies (Round Rock, TX, USA) and the operating system of the computer was Ubuntu 18.04.

To obtain the point cloud in ROI, the preprocessing was realized by Loam_livox [36] and OPEN3D [37], which included four steps: (1) Random sample consensus (RANSAC) was adopted to detect and remove the point cloud of the ground (outdoor), ceiling, and walls (indoor). (2) Depth filtering was used to remove the point cloud that was too high or too far away. (3) Sparse filtering was adopted to remove the point cloud, which was probable noise data. (4) Downsampling was used to reduce the data size of the point cloud. The clustering process was realized by MATLAB R2020b.

To investigate the point cloud segmentation performance of the improved DPC algorithm, the basic DPC [15] and two classic unsupervised clustering algorithms (K-means and DBSCAN) were introduced for comparison. The number of cluster centers and the cut-off distance of the basic DPC were manually set ideally [15]. The K-means and DBSCAN were realized by using the functions “kmeans” and “dbscan” in the Statistics and Machine Learning Toolbox of MATLAB, respectively [1,2,3]. The number of clusters of K-means, the Eps (the radius of the neighborhood), and the MinPts (the minimum number of points in the neighborhood) of DBSCAN were also manually set to ideal numbers [1,2,3].

For the improved DPC algorithm, in the experiments, the side length of voxels $l_{s}$ was set as 0.1 m ($l_{s}=0.1\;m$). The $m_{0}$ (the number of $\gamma_{m}$ which were used for curve fitting) was set as 30 ($m_{0}=30$). The distance $\tilde{d}$ was set as 2 times the side length of voxels $l_{s}$ ($\tilde{d}=2l_{s}$). The number $N_{min}$ was set as 5 ($N_{min}=5$).

The accuracy (*Acc*), the adjusted rand index (*ARI*) and the computation time were used as indexes to evaluate the clustering performance [1,2,3,25]. The *Acc* was defined as:

(10) $$Acc=\frac{N_{correct}}{N},$$

where $N_{correct}$ was the number of data points that were correctly segmented. *N* was the total number of all data points. *Acc* ranges from 0 to 1. The value of *Acc* is expected to be higher when the clustering result is better.

The *ARI* was defined as:

(11) $$ARI=\frac{2\left(TP\cdot TN-FN\cdot FP\right)}{\left(TP+FN\right)\left(FN+TN\right)+\left(TP+FP\right)\left(FP+TN\right)},$$

where *TP* denotes the number of data point pairs that are in the same cluster under both the correct segmentation and the obtained segmentation; *TN* denotes the number of data point pairs that are in different clusters under both the correct segmentation and the obtained segmentation; *FN* denotes the number of data point pairs that are in the same cluster under the correct segmentation but in different clusters under the obtained segmentation; *FP* denotes the number of data point pairs that are in different clusters under the correct segmentation but in the same cluster under the obtained segmentation. *ARI* ranges from −1 to 1. The value of *ARI* is expected to be higher when the clustering result is better.

### 4.2. Experimental Results and Discussion

**Figure 7:**
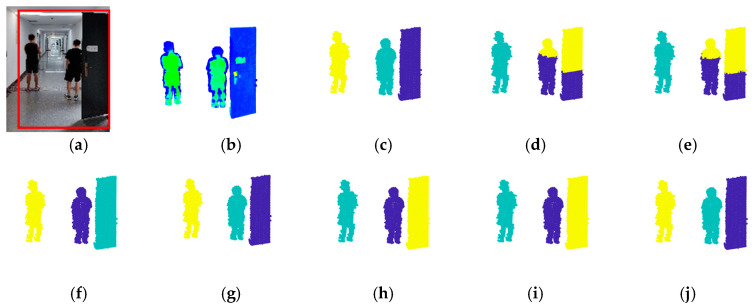
Experimental results of Scene 1. (**a**) The photo of the scene. (**b**) The point cloud of ROI. (**c**) The correct segmentation. (**d**) K-means. (**e**) Voxel + K-means. (**f**) DBSCAN. (**g**) Voxel + DBSCAN. (**h**) DPC. (**i**) Voxel + DPC. (**j**) The new improved DPC.

**Figure 8:**
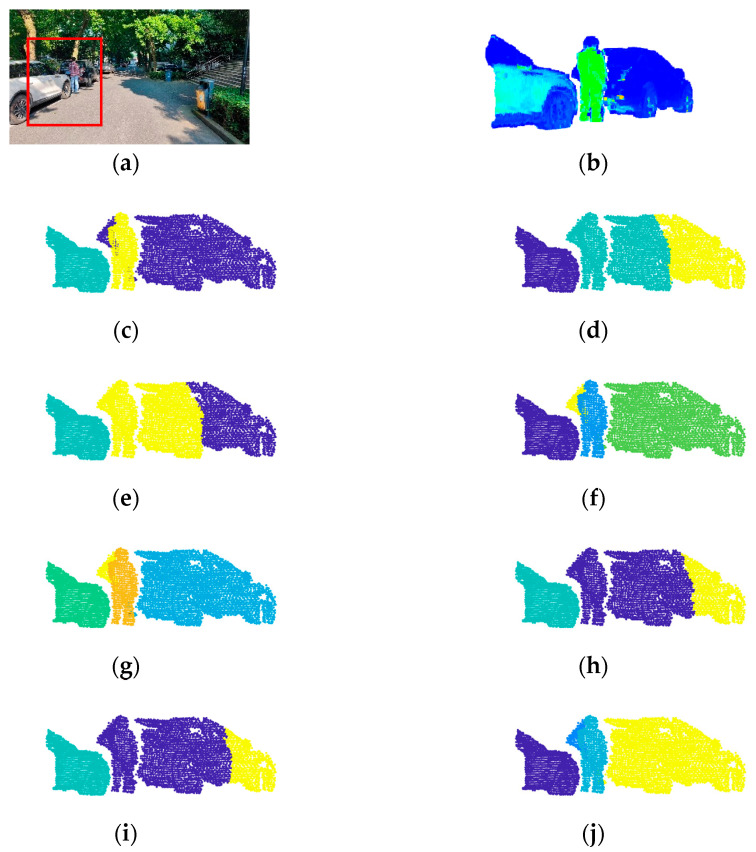
Experimental results of Scene 2. (**a**) The photo of the scene. (**b**) The point cloud of ROI. (**c**) The correct segmentation. (**d**) K-means. (**e**) Voxel + K-means. (**f**) DBSCAN. (**g**) Voxel + DBSCAN. (**h**) DPC. (**i**) Voxel + DPC. (**j**) The new improved DPC.

**Figure 9:**
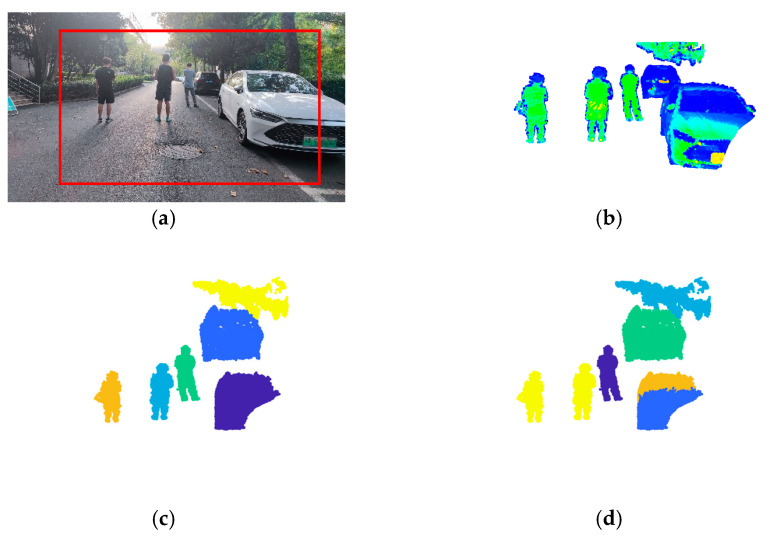

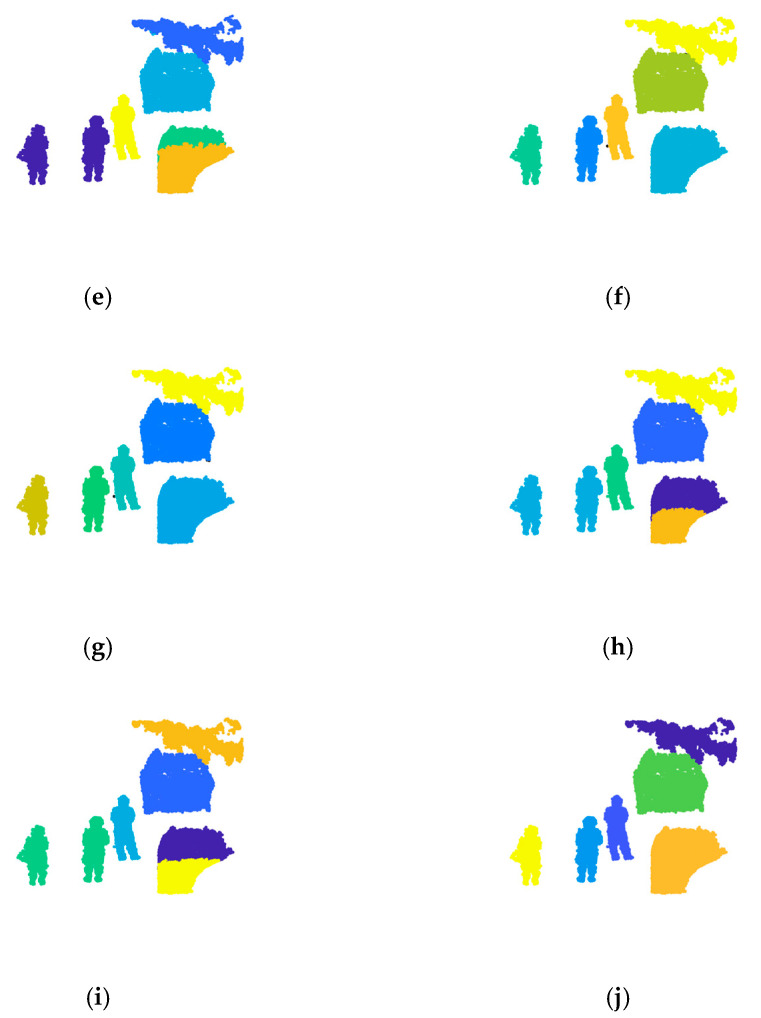
Experimental results of Scene 3. (**a**) The photo of the scene. (**b**) The point cloud of ROI. (**c**) The correct segmentation. (**d**) K-means. (**e**) Voxel + K-means. (**f**) DBSCAN. (**g**) Voxel + DBSCAN. (**h**) DPC. (**i**) Voxel + DPC. (**j**) The new improved DPC.

Figure 7, Figure 8 and Figure 9 show the experimental results.

The scene of Figure 7 (Scene 1) is a corridor with a door open and two people. The number of points in ROI is 2811, and the number of objects is 3. The scene in Figure 8 (Scene 2) is a road with two cars parked and one person standing between them. The number of points in ROI is 6197, and the number of objects is 3. The scene of Figure 9 (Scene 3) is relatively more complicated, which is a road with two cars parked, three people in the road, and a tree branch. The number of points in ROI is 12,444, and the number of objects is 6. The complexity of the three scenes (Scene 1, Scene 2, and Scene 3) are progressively increased to verify the performance of the improved DPC in both simple scenes and complex scenes. ROI is framed by red lines in each figure. Meanwhile, to further investigate the effectiveness of voxel structure, the point cloud segmentation results with voxel structure and without voxel structure are both provided. For Figure 7, Figure 8 and Figure 9, each figure has 10 pictures which are: (a) the photo of the scene with ROI framed by red lines, (b) the point cloud of ROI, (c) the correct point cloud segmentation result, (d) the point cloud segmentation result by K-means, (e) the point cloud segmentation result by K-means with voxel structure (Voxel + K-means), (f) the point cloud segmentation result by DBSCAN, (g) the point cloud segmentation result by DBSCAN with voxel structure (Voxel + DBSCAN), (h) the point cloud segmentation result by the basic DPC [15], (i) the point cloud segmentation result by the basic DPC with voxel structure (Voxel + DPC), (j) the point cloud segmentation result by the new improved DPC algorithm.

### 4.3. Discussion

Table 1 shows the comparison results of *Acc* under three scenes. Table 2 shows the comparison results of *ARI* under three scenes. Table 3 shows the comparison results of computation time under three scenes. The total number of points in each scene is listed after the name of it. The best result(s) in each row are highlighted by using the bold form.

The experimental results show that compared with the basic DPC (without voxel structure and with voxel structure), the improved DPC has the obvious advantages of higher accuracy and higher computation speed. Meanwhile, the improved DPC can realize the segmentation automatically without manual intervention. That means the improvement of DPC is successful. The proposed new approach to obtain $\rho_{i}$ (which is forming a voxel structure and using the number of points in the voxel as the local density of the voxel) and the proposed new approach to select cluster centers automatically (which is selecting the voxels whose gamma values are greater than the gamma value of the inflection point of the fitted γ curve as cluster centers) are effective approaches to avoid the cut-off distance and select the cluster centers automatically. The proposed new merging strategy is also effective. The new merging strategy can overcome the over-segmentation problem and obtain the point cloud segmentation results successfully.

The experimental results also show that compared with K-means (without voxel structure and with voxel structure), the new method has the obvious advantage of higher accuracy. That is because DPC is effective to process non-spherical data, and the proposed automatic selection of cluster centers and the new merging strategy can further improve the clustering accuracy. While K-means tends to obtain spherical clusters. Compared with the accuracy of DBSCAN (especially DBSCAN with the proposed voxel structure), the accuracy of the improved DPC is comparable or slightly better. DBSCAN and DPC are both density-based clustering algorithms. So, it is reasonable that the results are comparable under some scenes, such as Scene 1 and Scene 2 in this work. However, DBSCAN may mis-regard some data points as extra noise data, which reduces the accuracy slightly, such as Scene 3 or some objects with low density points. Additionally, choosing appropriate threshold parameters for DBSCAN can be nontrivial [15].

The experimental results also indicate that compared with the K-means and DBSCAN (whether with voxel structure or not), the speed of the improved DPC is not satisfactory. The research results are in accord with the theoretical analysis of DPC [16,17,18,19,20,21,22,23,24,25,26,27], because although the improved DPC is faster, its intrinsic time complexity has not been changed. Research works have verified that the time complexities of K-means, DBSCAN, and DPC are $O\left(N\right)$, $O\left(N^{2}\right),$ and $O\left(N^{2}\right)$, respectively [1,2,3]. That shows the time complexity of K-means is lower than DPC, while the time complexities of DBSCAN and DPC are the same. Actually, the time complexities of DBSCAN and DPC are both $O\left(N^{2}\right)$. However, in this work, the DBSCAN is implemented by directly using the “dbscan” function in MATLAB, where the algorithm is concisely programmed and the K-dimensional tree is used to accelerate the algorithm with the time complexity of $O\left(NlogN\right)$. While the improved DPC is programmed by the authors. These result in that the speed of the DBSCAN is faster than that of the improved DPC.

## 5. Conclusions

In this work, a new improved DPC algorithm is proposed. Unlike the conventional DPC algorithm, the improved DPC algorithm can process the clustering automatically without manual intervention.

To assess the clustering performance of the improved DPC algorithm, experiments on point cloud segmentation of LiDAR under different scenes were carried out in the real world. The experimental results verify the effectiveness of the improved DPC algorithm. Compared with the conventional unsupervised clustering algorithms, the improved DPC algorithm has the advantage of higher accuracy.

The research work provides an effectively improved DPC algorithm and extends the application fields of DPC, which provides a useful reference for others’ research work. The results have indicated that using DPC can effectively realize high-accuracy point cloud segmentation and have shown the great developing potential of DPC-based algorithms in the fields of obstacle detection, simultaneous localization and mapping (SLAM) and navigation, etc. However, DPC has the disadvantage of relatively high time complexity, which needs further improvement. How to further improve the DPC and reduce the intrinsic time complexity of the DPC is meaningful research work to be undertaken in the future.

## Figures and Tables

**Table 1:** The Comparison Results of *Acc* under Three Scenes.

	K-Means	Voxel + K-Means	DBSCAN	Voxel + DBSCAN	DPC	Voxel + DPC	New Method
Scene 1 (2811)	0.756	0.751	**1.000**	**1.000**	**1.000**	**1.000**	**1.000**
Scene 2 (6197)	0.560	0.612	**0.963**	**0.963**	0.715	0.747	**0.963**
Scene 3 (12,444)	0.731	0.745	0.997	0.997	0.834	0.800	**0.998**

**Table 2:** The Comparison Results of *ARI* under Three Scenes.

	K-Means	Voxel + K-Means	DBSCAN	Voxel + DBSCAN	DPC	Voxel + DPC	New Method
Scene 1 (2811)	0.486	0.480	**1.000**	**1.000**	**1.000**	**1.000**	**1.000**
Scene 2 (6197)	0.430	0.438	**0.912**	**0.912**	0.472	0.500	**0.912**
Scene 3 (12,444)	0.716	0.717	0.990	0.993	0.785	0.747	**0.997**

**Table 3:** The Comparison Results of Computation Time under Three Scenes. (Unit: s).

	K-Means	Voxel + K-Means	DBSCAN	Voxel + DBSCAN	DPC	Voxel + DPC	New Method
Scene 1 (2811)	0.080	**0.002**	0.030	0.003	0.535	0.017	0.014
Scene 2 (6197)	0.085	**0.004**	0.078	0.009	2.721	0.125	0.068
Scene 3 (12,444)	0.104	**0.014**	0.202	0.033	12.679	0.629	0.376

## Data Availability

The data presented in this study are available on request from the corresponding author. The data are not publicly available due to protection of intellectual property.
